# Outcome of the elderly patient in intensive care: a cohort study

**DOI:** 10.1186/2197-425X-3-S1-A531

**Published:** 2015-10-01

**Authors:** AD Impiumi

**Affiliations:** Università degli Studi di Padova, Padova, Italy

## Introduction

The world population is ageing, by 2050 the percentage of the population older than 80 years will double. Older age is associated with higher prevalence of chronic illness and functional impairment, contributing to an increased rate of hospitalization and admission to Intensive Care Units.

## Objectives

To assess if age is an important and independent predictor of the mortality rate of patients of 80 years old and older who are admitted to the Intensive Care Unit.

## Methods

Retrospective cohort study. We included all patients admitted to the polivalent Intensive Care Units ISTAR 1 and ISTAR 2 in 2011 and 2013 until June. Patients were categorized into three age groups: less than 80 years old, 80-85 years old and above 85 years old. Age, type of admission, comorbidities, hospital outcome and duration of recovery were analyzed. Bivariate and multivariate analysis were used to analyze age, type of admission (surgery versus medical versus emergency) and comorbidities' influence on hospital outcome. Kruskal-Wallis test was used to compare duration of recovery in our three age groups. Kaplan-Meier survival curves of the three age groups were designed and compared with the log-rank test.

## Results

Age, as a standalone factor, is a significant predictor of mortality rate among patients admitted to the Intensive Care Unit (p = 0.0290). The type of admission is even more strongly associated with mortality (p < 0.0001). BPCO (p = 0.0446) and cardiopathy (p = 0.0028) are significant predictor factors of hospital outcome, while cancer is not (p = 0.5676).

## Conclusions

Age resulted to be a significant predictor of mortality rate, but not as much as COPD and cardiopathy, that seem to be better predictors. Age does not appear to be the only criteria upon which evaluate the aptness to admit a patient to the Intensive Care Unit.

## Grant Acknowledgment

From this study we have learnt that age must be taken into account when deciding whether or not to admit a patient in Intensive Care but with all the other variables such as comorbidities and type of admission, which are shown to be even more significant than age itself to predict the outcome of the patient.
